# Acute impact of light at night and exogenous melatonin on subjective appetite and plasma leptin

**DOI:** 10.3389/fnut.2022.1079453

**Published:** 2022-12-06

**Authors:** Mohammed S. Albreiki, Ghalia H. Shamlan, Ahmed S. BaHammam, Nawaf W. Alruwaili, Benita Middleton, Shelagh M. Hampton

**Affiliations:** ^1^Center for Biotechnology, Khalifa University, Abu Dhabi, United Arab Emirates; ^2^Centre for Chronobiology, Faculty of Health and Medical Sciences, School of Bioscience and Medicine, University of Surrey, Guildford, United Kingdom; ^3^Department of Human Nutrition, College of Food Science and Agriculture, King Saud University, Riyadh, Saudi Arabia; ^4^National Plan for Science and Technology, College of Medicine, King Saud University, Riyadh, Saudi Arabia; ^5^The University Sleep Disorders Center, College of Medicine, King Saud University, Riyadh, Saudi Arabia; ^6^Department of Community Health Sciences, College of Applied Medical Sciences, King Saud University, Riyadh, Saudi Arabia

**Keywords:** metabolism, melatonin, light at night, leptin, appetite

## Abstract

This study investigates the possible effect of exogenous melatonin on appetite control by investigating plasma leptin and subjective appetite parameters. Nine healthy male participants [26 ± 1.3 years, body mass index (BMI) 24.8 ± 0.8 kg/m^2^] (mean ± SD) were recruited. The study was designed as a randomized three-way cross-over design; light (>500 lux) (LS), dark (<5 lux) + exogenous melatonin (DSC), and light (>500 lux) + exogenous melatonin (LSC), with an interval of at least 7 days between each session. Each session started at 18:00 h and ended at 06:00 h the following day. Participants were awake and in a semi-recumbent position during each clinical session. The meal times were individualized according to melatonin onset from 48 h sequential urine collection, whereas melatonin intake was given 90 min before the evening meal. Subjective appetite parameters were collected at 30 min intervals during each session. Plasma leptin was collected at specific time points to analyze pre-prandial and postprandial leptin. Subjective hunger and desire to eat were reported higher in LS than DSC and LSC (*P* = 0.03, and *P* = 0.001). Plasma leptin showed a significant increase in LSC and DSC (*p* = 0.007). This study suggested a positive impact of exogenous melatonin on subjective appetite and plasma leptin.

## Introduction

Artificial light at night (ALAN) has grown exponentially over modern societies’ natural nocturnal lighting levels. Although ALAN has provided substantial benefits to humankind, the adverse biological impacts of ALAN have been widely investigated (1–3). Extensive studies have been conducted to understand ALAN’s mechanism and health hazards against human physiology. The link between ALAN and disruption of circadian rhythm has been well established (4, 5), which can contribute to alterations in metabolism (6, 7), and increased risk of chronic disorders such as obesity and type 2 diabetes (8). Furthermore, light can directly affect endocrine signaling from circadian dysregulation or impaired melatonin production (9).

Melatonin has been associated to various biological processes due to its widely distributed melatonin receptors. Various studies have linked melatonin to lipid and glucose metabolism (10, 11), vascular function (12), appetite (13), and behaviors (14). Therefore, melatonin suppression as a result of ALAN may potentially have an impact on metabolism. Melatonin acts as a hormonal mediator of photoperiodic information, regulating energy homeostasis by balancing energy intake and energy expenditure. Add to this, the diversity of melatonin binding sites in gastrointestinal tracts suggest various possible function of melatonin in appetite regulation.

Leptin is a hormone that is mainly released by adipose tissue to maintain energy balance and appetite regulation. Melatonin’s role in the release of leptin has been highlighted in several animal studies. The administration of melatonin in middle-aged goldfish and rats results in a decrease in plasma leptin levels, and this impact was inverted in goldfish by the administration of a melatonin antagonist (15, 16). Other contradictory results revealed that consistent light exposure in rats could increase food intake consumption (17) or abolish day/light variation of leptin levels (18). Despite the controversial results in animal studies, the majority of studies suggested that melatonin administration may increase the circulating leptin levels (19). The role of melatonin in controlling appetite and weight has been discussed by Buonfiglio et al. (20) showing that deletion of melatonin receptor type 1 in the hypothalamus was associated with the malfunction of leptin signaling and leptin resistance (20).

There are few and controversial findings from human studies that discuss the impact of melatonin on appetite hormones such as leptin and ghrelin. Figueiro et al. (21) reported a boost in leptin levels and a drop in ghrelin in sleep-restricted individuals after morning light exposure (21). Similarly, an increased sense of hunger (22, 23), and reduced leptin (24, 25) were associated with sleep restriction or sleep debt. A recent study has shown a reduced body weight and body mass index (BMI) among overweight night shift workers after melatonin supplementation (26), whereas eating habits among female night shift workers with excessive weight have not changed after melatonin administration (27). In addition, a recent meta-analysis included seven clinical trials and 244 cases did not support the melatonin impact on body weight and appetite (28). We have previously shown that exogenous melatonin/ALAN was associated with reduced glucose tolerance, insulin insensitivity, and changes in lipid profile among healthy young males (10). We hypothesize that exogenous melatonin can alter subjective appetite and plasma leptin. Therefore, this study was conducted to assess the effects of exogenous melatonin administration on subjective appetite and plasma leptin among healthy young males.

## Methods

### Ethic statement and recruitment

The ethics committee at the University of Surrey approved all parts of the study (UEC/2015/021/FHMS). The methodology and experimental design were discussed during an induction session for participants who met the inclusion and exclusion criteria (Supplementary material A). The chosen participants signed a written consent form before attending the clinical session, confirming they were aware of the potential hazards and discomforts. All included participants were a young male student from University of Surrey (Guildford, UK). All participant information was tagged and rigorously kept in accordance with the Data Protection Act (1998).

### Screening procedures

Participants were required to complete multiple questionnaire such as general health questionnaire, Horne-Ostberg (HÖ) evaluation, Pittsburgh sleep quality index (PSQI), Munich chronotype questionnaire (MCTQ), and daily sleep diary. HÖ was completed to assess the chronotype of participants (29). PSQI evaluates the sleep quality assessment for all participants using PSQI was evaluated (30), whereas Munich chronotype was completed to evaluate sleep schedule during working/free days (31). Before the clinical session, participants wore Actiwatches to track their sleep-wake cycle. Furthermore, participants were not on night duties, or crossed more than 2 time zones in the month prior to the study session.

Caffeinated beverages, alcohol, strenuous activity, and analgesic medicine usage were prohibited for 24 h prior to the laboratory sessions. In addition, cosinor analysis was used to determine acrophase of 6-sulfatoxymelatonin (aMT6s) (Stockgrand Ltd., University of Surrey, Guildford, UK), by analyzing a 48-h sequential urine collection from all participants. This will assist in determining the rising phase of participants’ endogenous melatonin, thus allowing meal intakes (supper) to be individually assigned for each participants (Supplementary materials B,C). Participants who met the inclusion and exclusion criteria, plus passing the screening procedures were allowed to join the clinical sessions.

### Meal and circadin timings

Table 1 shows the composition and macronutrients of meals served during the clinical session. Breakfast was served at 08:00 h, whereas lunch and the evening meal “supper” were tailored to the acrophase time of urinary aMT6s. All meals were served at the Clinical Investigation Unit, University of Surrey. The average fasting period between lunch and the super was 9–10 h. Circadin prolonged-release melatonin (Neurim Pharmaceuticals Ltd.) containing 2 mg melatonin was utilized to maintain increased melatonin levels for 8–10 h (32). Circadin was administered orally 90 min before the supper to ensure sufficient melatonin levels throughout the session (Supplementary material B).

**TABLE 1:** Carbohydrate, protein, fat, fiber, and energy for each of the meals and overall composition of all three meals.

Meal/g	Energy Kcal	Protein (g)	CHO (g)	Fat (g)	Fiber (g)
Breakfast	627	15	98	16	14
Lunch	927	25	115	38	19
Test meal “Supper”	1,066	38	104	54	7
Total	2,620	78	317	105	40
% composition*		15%	59%	19%	7%

*Percentages were calculated proportionally from the total daily consumption.

### Study session

**FIGURE 1:**
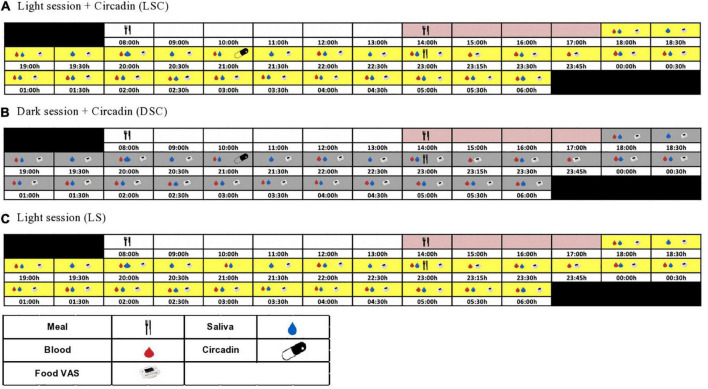
Study protocol. The schematic figure represents the study protocol for a participant with plasma melatonin onset at 20:30 h. All interventions (see key) were relative to each participants’ melatonin onset.

Participants were randomized using a three-way cross-over design protocol: light session (LS) (>500 lux), light + Circadin session (LSC) (>500 lux) and dark + Circadin session (DSC) (<5 lux), with at least 7-day washout period. Each session began at 18:00 h and ended at 06:00 h the next day. Participants were assigned to one of two groups: A or B. Group A attended LS first, followed by DSC and then the LSC session, whereas Group B started with LSC first, followed by DSC and then the LS session (Supplementary material D). Sequencing effects were statistically evaluated in the data. Figure 1 depicts the study protocol procedures. During the investigation, both body movement and posture were rigorously controlled. The participants were instructed to remain semi-recumbent (being at around 45°). They were also allowed to use the toilet following sample collection. Nonetheless, they had to rest in a semi-recumbent position for around 15 min before the next sample collection to ensure that all participants’ bodily motions and energy consumption were consistent during the study sessions. The light intensity and irradiance were recorded and maintained during the three sessions. The Supplementary Table E. 1 shows total photon flux (photons/cm2/cm), light intensity (lux), and irradiance (w/m2).

At 08:00 h, each laboratory session began with breakfast. Participants were allowed to leave the CIU after breakfast, but to return at lunch time. After lunch, all participants were asked to stay at the CIU until the clinical session starts. The lunch and late evening test meals were tailored to the estimated melatonin onset from urinary aMT6 acrophase timing. Circadin was given 90 min before the late evening test meal in the DSC and LSC sessions to ensure that melatonin levels remained elevated throughout the session. Plasma leptin was collected hourly, beginning at 18:00 h and continuing until the late evening test meal. The samples were taken every 15 min for the first hour following the meal, then every 30 min until the session was over. The meal VAS questionnaire was recorded using a visual analogue scale (VAS PRO-Diary) at 30 min intervals throughout the session (Figure 1). VAS-Pro-Diary measured three types of subjective appetite: hunger, desire to eat, and fullness. Human leptin radioimmunoassay kit from Millipore Company (Billerica, MA, USA) was used to measure plasma leptin. The inter-assay and coefficient variation for plasma leptin was 4.1% for low-level quality control and 6.7% for high-level quality control. In contrast, Intra-assay for low and high-level quality controls were 5.5 and 4.2%, respectively.

### Statistical analysis

Based on data from a prior investigation (7), a power calculation was performed using PS software (Vanderbilt University, Nashville, TN, US) with a power of 80% and a significance level of 0.05. This power estimate indicated that 12 or more participants were required; however, data sets were only retrieved from *n* = 9. The cosinor analysis to determine the peak of aMT6s was performed using a built-in calculation developed by Dr. D S Minors at University of Manchester (Supplementary material F).

Normality test using the D’Agostino-Pearson omnibus was assessed (GraphPad, San Diego, CA, USA). Mean, standard deviation, and standard error were calculated for all data. All hormonal and metabolic data were subjected to three-factor repeated measures (light, melatonin, and time) ANOVA, followed by Tukey’s honest significance test using statistical analysis software (SAS) software SAS Institute Inc., Cary, NC, USA. The total area under the curve (TAUC) and Incremental area under the curve (IAUC) were calculated using the trapezoidal and incremental rule (TAUC and IAUC). TAUC and IAUC were used to examine the hormonal and metabolic data, which was then followed by one-way ANOVA and Tukey’s multiple comparison testing. The level of significance was fixed at *p* ≤ 0.05.

## Results

Twelve participants were recruited for this study, and only nine completed the study. All nine participants were males with an average age of 26 ± 1.3 and BMI of 24.8 ± 0.8 (Table 2). Participants’ demographics, such as smoking and caffeine consumption, are shown in Table 2. The average score of PSQI was 3.5 ± 0.4, indicating good sleep quality, whereas HÖ scored 54.5 ± 2.6. In addition, three participants were classified as moderate morning type, whereas the remaining six were neither morning nor evening type. The mid-sleep time during the free days ranged between 03:30 to 06:30 h, and the average of all participants was 04:46 ± 00:22. Sleep parameters analyzed by Actiwatches showed no significant difference in sleep latency, efficiency and fragmentation index prior to each session (Supplementary material G). Participants have a sleep duration of approximately 6 h, with sleep efficiency above 70% and a fragmentation index over 40.

**TABLE 2:** Participant demographics.

	All participants (*n* = 9) (mean ± SEM)
Age (year)	26 ± 1.3
Body weight (Kg)	75.3 ± 3.1
Height (m)	1.7 ± 0.02
BMI (kg/m^2^)	24.8 ± 0.8
Caffeine/wk	6.2 ± 2.5
Alcohol/wk	0.2 ± 0.2
PSQI^a^	3.5 ± 0.4
H*Ö^a^*	54.5 ± 2.6
MCTQ^a^ (h)	04:46 ± 00:22
RBC^a^ (10^3^/mm^3^)	5.1 ± 0.1
WBC^a^ (10^3^/mm^3^)	5.6 ± 0.4
PLT^a^ (10^3^/mm^3^)	235 ± 11.7
HGB^a^ (g/dl)	13.8 ± 0.3

Values are mean ± SEM. RBC, red blood cell; WBC, white blood cell; PLT, platelet; HGB, hemoglobin.

^a^Values given are those obtained during the screening session.

### Subjective appetite scores

**FIGURE 2:**
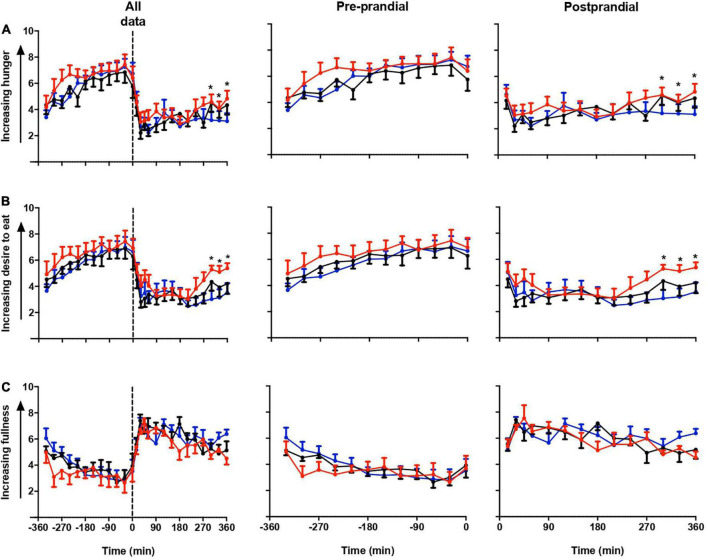
Subjective appetite parameters. Subjective hunger **(A)**, desire to eat **(B)**, and fullness **(C)** ratings (mean ± SEM) prior to and after a standard evening meal (time = 0 black dotted line) during LS (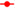), DSC (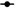), and LSC (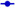) in all participants (*n* = 9) (*T* = –330 to *T* = +360 min).

Figure 2 shows the repeated measure ANOVA of subjective hunger, desire to eat and fullness score in all three sessions (Figure 2). Participants felt more hunger and desire to eat in LS than in DSC and LSC (*P* = 0.03, and *P* = 0.001), respectively, whereas no significant difference was shown in the fullness score (*P* = 0.9). The significant differences were only reported at postprandial times (+270, +300, +330, and +360 min). The significant effects of times were reported in all three subjective parameters during all three sessions (*P* < 0.001).

### Pre-prandial and postprandial of plasma leptin

**FIGURE 3:**
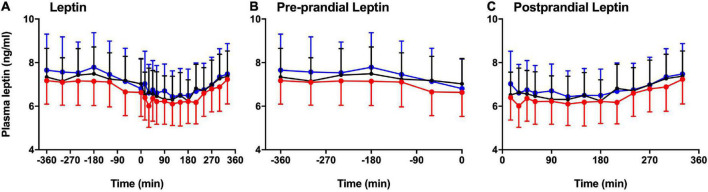
Pre-prandial and postprandial plasma leptin. Pre-prandial and postprandial plasma leptin (mean ± SEM) levels prior to and after a standard evening meal (time = 0 black dotted line) during LS (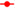), DSC (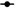), and LSC (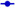) in all participants (*n* = 9). **(A)** Plasma leptin between –360 to +330 min, **(B)** pre-prandial leptin between –360 to 0 min, and **(C)** postprandial leptin between +15 to +330 min [*F_(1,496)_* = 11.54, *p* = 0.007].

**FIGURE 4:**
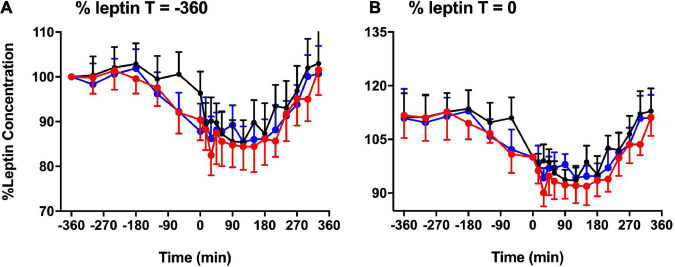
Basal leptin concentration. % basal leptin (mean ± SEM) levels directly prior to the test meal (*T* = 0) **(A)** and at time –360 **(B)** during LS (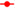), DSC (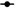), and LSC (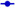) (*n* = 9) (*T* = –360 to *T* = +330 min) [*F*_(1,496)_ = 11.54, *p* = 0.007].

Figure 3 shows the significant difference in plasma leptin during pre-prandial and postprandial times. There was a significant effect of melatonin (*p* = 0.007) and time (*p* < 0.001) between the three session. Plasma leptin showed significant increase in LSC and DSC compared to LS (*p* = 0.007). Significant differences were spotted at the postprandial time at +210 and +330 min (Figures 3A–C). Better visualization of leptin response can be seen when the data were plotted as a % leptin of *T* = 0 or *T* = −360 (Figures 4A–B). Further details about the plasma leptin for all participants are shown in Supplementary material I.

### Total area under the curve and incremental area under the curve of plasma leptin

**FIGURE 5:**
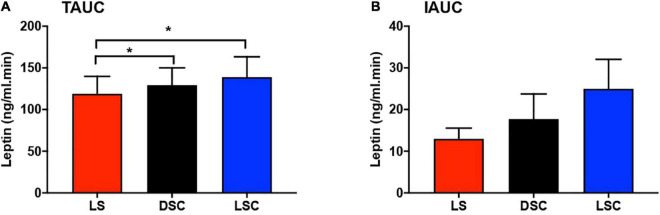
Total area under the curve (TAUC) and incremental area under the curve (IAUC) of plasma leptin. TAUC **(A)** and IAUC **(B)** for plasma leptin (mean ± SEM) during LS (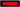), DSC (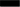), and LSC (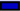) in all participants (*n* = 9) [TAUC: *F*_(1.5,12.1)_ = 7.06, *p* = 0.01; IAUC: *F*_(1.9,15.3)_ = 2.28, *p* = 0.13]. * Means *p* < 0.05.

Total area under the curves and IAUCs of plasma leptin are presented in Figures 5A–B. Plasma leptin was considerably higher in DSC and LSC than LS (*p* = 0.01). The Tukey’s multiple comparison testing showed the significant differences between LS and DSC (*p* = 0.04) and between LS and LSC (*p* = 0.01). TAUCs of plasma leptin in LS were 137.9 ± 21.1, 143.6 ± 22.2 in DSC, and 146.7 ± 27.5 ng/ml.min in LSC. No significant differences were reported in IAUC between all three session (*p* = 0.13). IAUC of plasma leptin in LS was 15.5 ± 2.5, 25.1 ± 7.1 in DSC, and 28.2 ± 8.1 ng/ml.min in LSC. Further details about the TAUC and IAUC of plasma leptin for all participants are shown in Supplementary materials J–K.

## Discussion

This study investigated the effect of exogenous melatonin on plasma leptin as the primary appetite hormone was investigated. This is the first study to assess the acute effect of exogenous melatonin on subjective appetite score and plasma leptin among healthy young males. This is part of a large clinical study that investigated the impact of exogenous melatonin/ALAN on hormones and metabolites (10). Data from recent studies revealed the presence of contradictory results on melatonin-leptin interactions. In some mammals, melatonin administration has increased leptin levels (33, 34), whereas leptin was decreased in middle-aged rats and goldfish (15, 16, 19). However, these factors influenced the above controversies due to: (1) Different species; (2) Melatonin had adverse action on the adrenal glands, which triggered changes in glucocorticoids levels (35), or elevated the hypothalmo-pituitary-adrenal axis as result of pinealectomy (36); and (3) leptin suppression due to weight reduction (15, 37). Add to this, the possible differences in the physiological meaning of melatonin between nocturnal and diurnal animals could explain the discrepancy in the melatonin-leptin results reported in animal and human studies.

Consequently, our results revealed a notable increase in leptin level with melatonin administration because leptin was significantly higher in DSC and LSC than in LS. Eventually confirmed by TAUC and IAUC, this rise was quite evident when the data were split into “before and after meal periods” or “pre and post-prandial periods.” Contrarily, our study conflicts with that of Figueiro et al. (21), who reported that leptin levels increased after morning light exposure (21). It likewise contradicts Cheung et al. (38), who reported no changes in leptin or ghrelin levels after exposure to blue-enriched light (38). Investigating the melatonin levels would have shed more light on their findings but both studies did not do that. Besides, their study designs did not cover the melatonin profile window. This opens a question whether changes reported above were due endogenous melatonin exerting metabolic effects.

The expression and release of the leptin gene were stimulated by glucocorticoid administration (39). We previously investigated cortisol in this study, yet cortisol’s possible stimulatory effects were absent, as there were no apparent differences between the three sessions (10). Moreover, insulin-leptin interaction have been more controversial (40–42). Yet, Alonso-Vale et al. (43) showed that while insulin acted in synergy with melatonin to enhance the expression of the leptin gene, insulin or melatonin alone did not affect leptin gene expression. They further reported that through the Pertussis toxin (PTX)-sensitive Gi protein-coupled membrane receptor, melatonin could obstruct forskolin’s inhibitory effect on both leptin synthesis and secretion in adipocytes (43). Insulin signaling is involved in leptin expression *via* melatonin receptor type I (MTI), and melatonin could increase its signaling effect (43). Although with melatonin administration, insulin levels were much lower (10), the melatonin-insulin action upon adipocytes cannot be unnoticed.

It is likewise essential to note that endocrine and metabolic status can be aggravated during major depression (44, 45). A recent meta-analysis have indicated metabolic and inflammatory dysregulation were strongly associated with atypical depression (46). One example is the report that those in a depressed mood tend to take more food (47). However, in this study, participants reported no difference in depressive mood between the three sessions. Meanwhile, this hypothetical mechanism is at odds with the notable increase in the subjective miserable score during LSC compared to LS (Supplementary material H). Nonetheless, analyzing short-term appetite control associated with other appetite biomarkers like cholecystokinin (CCK) and ghrelin could be more helpful in this condition. According to Spiegel et al. (23), the effect of insomnia or sleep deprivation on leptin levels is well documented (23). Nevertheless, during all three sessions in this study, participants were sleep deprived. This implies that leptin changes are not really influenced by sleep deprivation.

In this study, with the opposite in fullness score, compared to DSC and LSC, we saw significant increases in subjective hunger and desire to eat scores in LS. Our data revealed a notable effect of melatonin administration on subjective appetite scores. The significant differences shown in plasma leptin are explained by subjective hunger and desire to eat. These results contradicts to that of Cheung et al. (38), who reported that after exposure to blue-enriched light administration, both in the morning and evening, there were no changes in subjective hunger (38). This opens a question of whether melatonin intake can reduce body weight as reported in early type chronotype night shift workers (26). Our plasma leptin and subjective parameters support these findings, yet it is noteworthy to indicate that the evening test meal was served in the same room during the three sessions. Therefore, the smell of food by subjects or subjects with earlier dim light melatonin onset (DLMO) who had their meals could potentially influence our food VAS results.

There was an evident melatonin suppression increase in LS, shown in subjective appetite scores for hunger and desire to eat. Melatonin administration in this study, strengthened the possibility of a melatonin-leptin interaction. So, most likely because of the lower plasma leptin, participants reported higher hunger and desire to eat. So, according to Alonso-Vale et al. (43), forskolin’s inhibitory effect on leptin synthesis and secretion in adipocytes is prevented by melatonin. Melatonin also increases the signaling power involved in insulin-induced leptin expression *via* MTI (43). Defined as the hormonal regulatory loop involving leptin-insulin interaction, never ignore the importance of the adipo-insular axis.

A key strength of the present study was the highly controlled in-laboratory and strict session monitoring of participants prior to attending the clinical session. For instance, melatonin intake and supper timing were individualized to ensure sufficient circulating melatonin when meal is given. A total of 9–10.5 h of fasting period was crucial to allow for sufficient collection for pre-prandial samples, and to ensure returning to basal fasting level at the beginning of the session. Light intensity and irradiance were well-controlled and monitored at the horizontal and vertical levels to ensure the same amount and correct intensity were delivered. Additionally, other non-photic factors capable of affecting melatonin, such as posture and calorie intake, were restricted. In contrast, there are multiple limitations that worth to be noted. This study was conducted only in males, with a low sample size. It is imperative to investigate the effect of melatonin in both genders and in a larger sample size to assess the significance level and minimize the limitations of statistical power. Various eating patterns as a result of adopting eating patterns, such as in athletes or amongst mixed cultural backgrounds, were not considered, which may influence the appetite analysis (48–50). The meals were served in the same room, thus the smell of food affecting the food VAS results cannot be ignored. Short-term appetite hormones such as ghrelin and CCK would add significant value to this study.

According to the major findings of this study, our data suggested a positive impact of exogenous melatonin on subjective appetite and plasma leptin. Our recent findings along with previously reported evidences demonstrated could possibly influence leptin release by modulating insulin levels (10). These findings also indicate the short-term effect of ALAN, which may become aggravated in long-term exposure, such as in shift work. Furthermore, helping to minimize obesity, the ability of melatonin to alter subjective hunger and desire to eat, plus influencing plasma leptin, may regulate food intake. Finally, although melatonin is available “over the counter” in some countries, it is noteworthy mention that the greater efficacy of MT1/MT2 receptor agonist may be effective for improving appetite control.

## Data availability statement

The raw data supporting the conclusions of this article will be made available by the authors, without undue reservation.

## Ethics statement

The studies involving human participants were reviewed and approved by the University of Surrey Ethics Committee (Study 1: EC/2013/93/FHMS and Study 2: UEC/2015/021/FHMS). The patients/participants provided their written informed consent to participate in this study.

## Author contributions

MA, BM, and SH designed the research. MA carried out the research and performed the data analysis. MA and GS wrote the manuscript. AB, NA, BM, and SH provided critical review. MA, GS, AB, BM, and SH have primary responsibility for the final content. All authors read and approved the final manuscript.
