# Supplementary material for: Acute impact of light at night and exogenous melatonin on subjective appetite and plasma leptin
Source: Front Nutr. 2022 Dec 6;9:1079453. doi: 10.3389/fnut.2022.1079453 (PMC9763572; doi:10.3389/fnut.2022.1079453)
Supplement: Supplementary file 1 [file Data_Sheet_1.docx]

**Supplementary A**

All participants were students at the University of Surrey. Strict inclusion and exclusion criteria were applied during recruitment. Participants who were eligible to take part in the study were provided with the study participant information sheet.

| The inclusion criteria were as follows:   - Aged between 18–60 years - BMI between 18.5–28.0 kg/m^2^ - Maintenance of selected regular sleep--wake cycle - Sleep duration between 6.5 to 8.0 hours - Must refrain from alcohol, caffeine, heavy exercise and carbonated drinks 24 hours before each session |
| --- |
| The exclusion criteria were as follows:   - Confirmed or possible pregnancy - Familial or personal history of psychiatric disorders - History of infectious conditions - History of drug or alcohol abuse. - History of taking any medication other than minor analgesics within 2 weeks prior to the study - Participation in any experimental drug trial within the last 3 months before the start of the study - Donation of blood within the last 3 months before the start of the study. |

**Supplementary B**

Table B.1 showed the average excretion rate and the amount of aMT6s excreted over 48 hours. Participants showed a substantial amount of aMT6s produced before joining the study as a part of screening procedures. Melatonin onset obtained from urinary aMT6s was compared with salivary melatonin onset which was calculated as described in Figure B.1. There were at least 1-hour differences between urinary melatonin onset and salivary melatonin onset.

Figure B.1: Calculation of meal timings


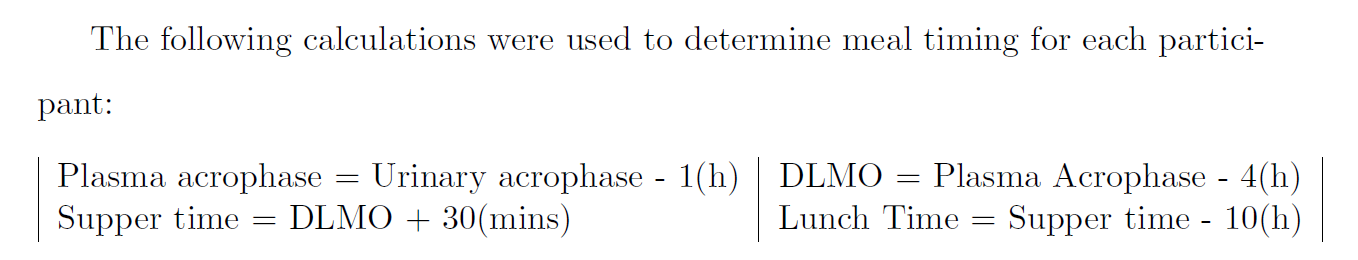


Table B.1: aMT6s urinary screening

| subject | Average aMT6s excretion rate  ng/h | | aMT6s mole | | S-melatonin onset | U-melatonin onset |
| --- | --- | --- | --- | --- | --- | --- |
| S001 | 1^st^ day | 390 | 1^st^ day | 2128 | 22:02 | 23:15 |
|  | 2^nd^ day | 464 | 2^nd^ day | 4211 |  |  |
| S002 | 1^st^ day | 1063 | 1^st^ day | 37933 | 20:32 | 22:45 |
|  | 2^nd^ day | 737 | 2^nd^ day | 23922 |  |  |
| S004 | 1^st^ day | 2021 | 1^st^ day | 39250 | 21:50 | 22:35 |
|  | 2^nd^ day | 1002 | 2^nd^ day | 26394 |  |  |
| S006 | 1^st^ day | 995 | 1^st^ day | 25736 | 20:35 | 22:00 |
|  | 2^nd^ day | 1073 | 2^nd^ day | 32043 |  |  |
| S007 | 1^st^ day | 767 | 1^st^ day | 39568 | 21:05 | 22:30 |
|  | 2^nd^ day | 1021 | 2^nd^ day | 40154 |  |  |
| S008 | 1^st^ day | 530 | 1^st^ day | 3490 | 22:30 | 23:00 |
|  | 2^nd^ day | 387 | 2^nd^ day | 11860 |  |  |
| S009 | 1^st^ day | 160 | 1^st^ day | 5895 | 23:00 | 23:45 |
|  | 2^nd^ day | 207 | 2^nd^ day | 5639 |  |  |
| S011 | 1^st^ day | 258 | 1^st^ day | 11105 | 23:00 | 00:45 |
|  | 2^nd^ day | 336 | 2^nd^ day | 19121 |  |  |
| S012 | 1^st^ day | 1323 | 1^st^ day | 24927 | 23:05 | 00:00 |
|  | 2^nd^ day | 667 | 2^nd^ day | 14822 |  |  |

**Supplementary C**

Table C.1: Plasma melatonin onset and meals timing of the participants in the study 2

| Subject | Acrophase aMT6s | Acrophase plasma | DLMO | Lunch | Supper |
| --- | --- | --- | --- | --- | --- |
| S001 | 04:15 | 03:15 | 23:15 | 14:45 | 23:45 |
| S002 | 03:45 | 02:45 | 22:45 | 14:15 | 23:15 |
| S004 | 03:35 | 02:35 | 22:35 | 14:00 | 23:00 |
| S006 | 03:00 | 02:00 | 22:00 | 13:30 | 22:30 |
| S007 | 03:30 | 02:30 | 22:30 | 14:00 | 23:00 |
| S008 | 04:00 | 03:00 | 23:00 | 14:30 | 23:30 |
| S009 | 04:45 | 03:45 | 23:45 | 15:00 | 00:00 |
| S011 | 05:45 | 04:45 | 00:45 | 15:15 | 01:15 |
| S012 | 05:00 | 04:00 | 00:00 | 15:00 | 00:30 |

**Supplementary D**

**Carryover Effect**

Data obtained from plasma melatonin have been used to examine the carryover “sequence” effect. This analysis was performed using Two-way RM ANOVA by testing the source of variations such as treatment, subject, sequence, and sequence x treatment.

Table D.1

| ANOVA Table | SS | DF | MS | P value |
| --- | --- | --- | --- | --- |
| Sequence x Treatment | 20.64 | 2 | 10.32 | 0.45 |
| Sequence | 1.215 | 1 | 1.21 | 0.85 |
| Treatment | 19.90 | 2 | 9.95 | 0.43 |
| Subject | 247.7 | 7 | 35.39 | 0.04 |

Figure D.1: Study flow chart


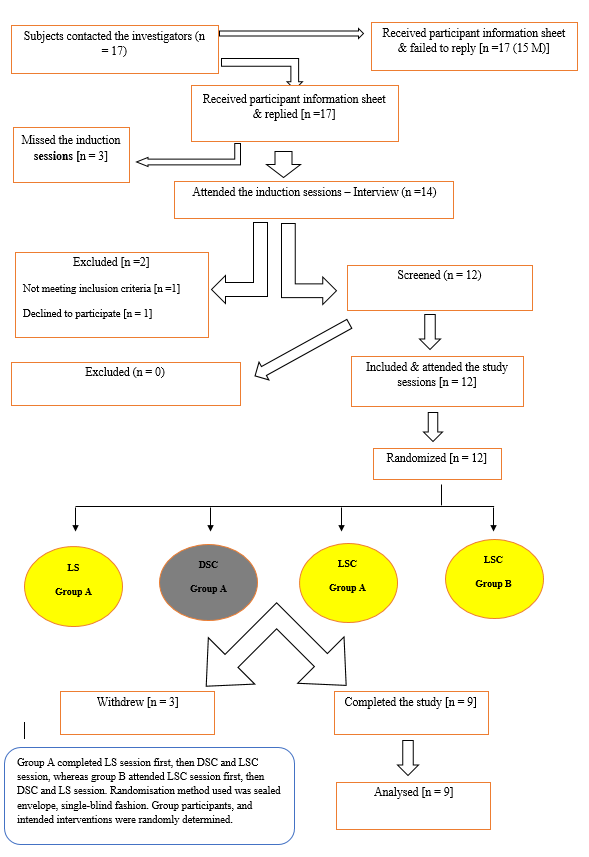


**Supplementary E**

Table E.1: Light measurements in LS, DSC and LSC

| Condition | Level | LS | DSC | LSC (n = 243) |
| --- | --- | --- | --- | --- |
| Light intensity lux | Horizontal (n = 243) | 563 ± 12 | 1.32 ± 0.2 | 548 ± 10 |
|  | Vertical (n = 243) | 342 ± 12.2 | 1.02 ± 0.03 | 350 ± 10.5 |
| Light irradiance w/m^2^ | Horizontal | 0.981 | 0.0012 | 0.981 |
|  | Vertical | 0.73 | 0.0008 | 0.73 |
| Photon flux photons/cm^2^/sec | NA | 3.12 x 10^19^ | 2.63 x 10^19^ | 3.12 x 10^19^ |

**Supplementary F**

Table F.1: Consinor analysis


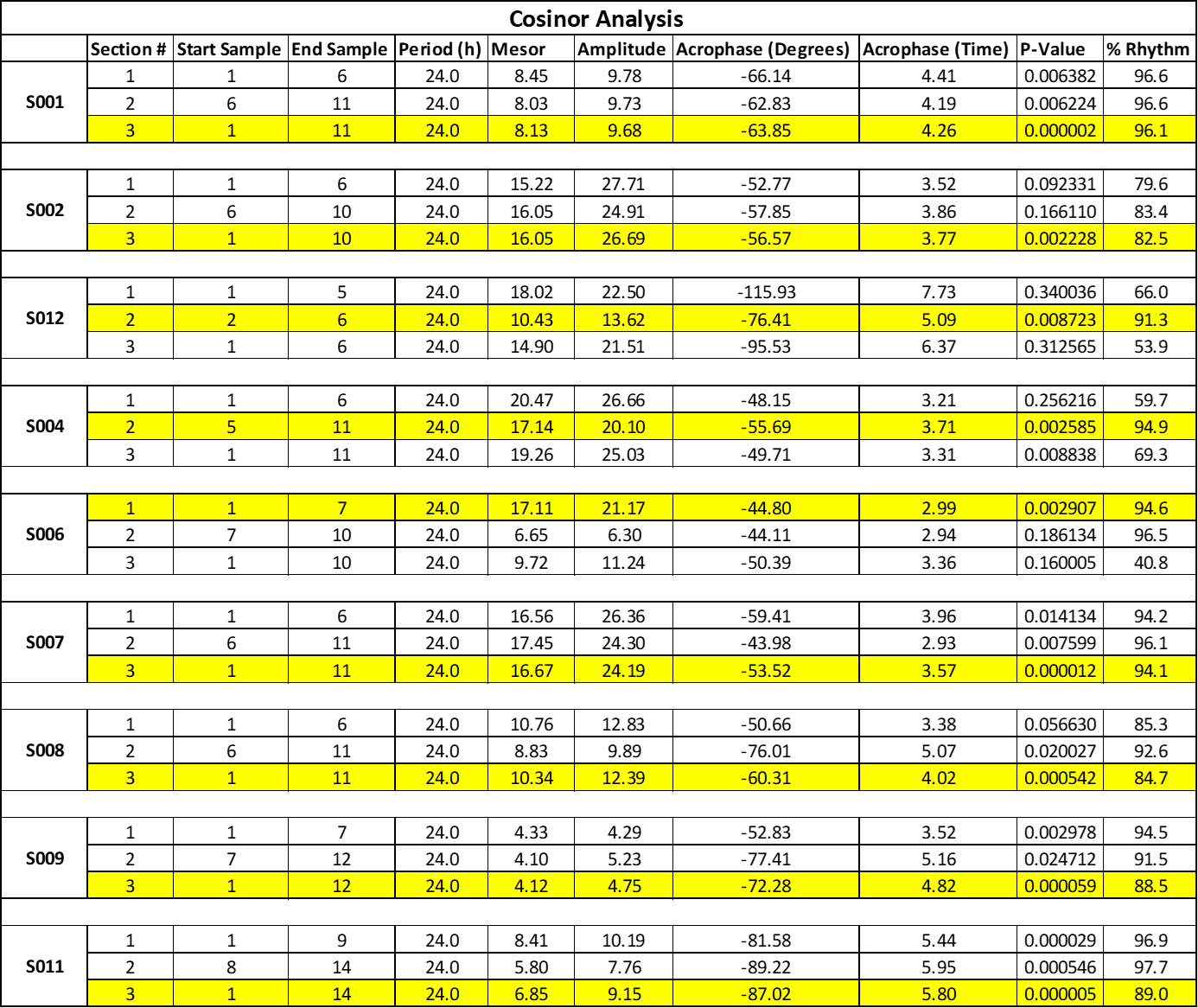


**Supplementary G**

Table I.1: screening sleep data

|  | LS | DSC | LSC | *P* |
| --- | --- | --- | --- | --- |
| Sleep start^a^, h:min | 24:40 ± 00:23 | 24:48 ± 00:16 | 24:51 ± 00:24 | 0.9 |
| Sleep end^a^, h:min | 07:47 ± 00:23 | 07:58 ± 00:23 | 07:57 ± 00:26 | 0.9 |
| Sleep duration^a^, h:min | 05:46 ± 00:14 | 05:53 ± 00:15 | 05:50 ± 00:19 | 0.9 |
| % Sleep efficiency^a^ | 77.4 ± 2.6 | 74.9 ± 2.5 | 75.9 ± 3.2 | 0.2 |
| Sleep latency^a^, h:min | 00:17 ± 00:03 | 00:42 ± 00:10 | 00:30 ± 00:14 | 0.1 |
| Fragmentation index^a^ | 41.48 ± 5.4 | 40.1 ± 5.1 | 41.2 ± 6.7 | 0.6 |

^a^Values are mean ± SEM, calculated by one-way ANOVA (n = 9). ^a^Values were obtained from 7 days prior to LS, DSC and LSC sessions.

**Supplementary H**

### Pre-prandial and postprandial KSS, mood and alertness

Schematic figure H.1 illustrates subjective KSS, alertness and moos scores at pre-prandial and postprandial times during LS, DSC and LSC. No significant differences were reported in KSS, alertness, mood2 and mood 3 (P = 0.2, P = 0.3, P = 0.3 and P 0.3) respectively. Participants were significantly feeling miserable in LSC compared to DSC and LS (P = 0.01). Post-hoc test revealed significant differences at test meal (T = 0) and at postprandial times (+30, +60, +180 and +210). All 5 subjective parameters showed significant effects of time (P < 0.001).

Figure H.1 Subjective mood and alertness

Subjective KSS (A), alertness (B), mood 1 (C), 2 (D) and 3 (E) (mean ± SEM) ratings prior to and after a standard evening meal (time = 0 black dotted line) during LS (
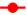
), DS (
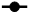
) and LSC (
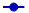
) in all participants (n=9).

**Supplementary I**

**Supplementary J**

**Supplementary K**
